# The Impact of Diabetes on Breast Cancer Development in Women: An Integrative Review

**DOI:** 10.1002/edm2.70276

**Published:** 2026-07-07

**Authors:** Israt Jahan, Mohammad Harun‐Ur‐Rashid, Imran Hossain, Fatema‐Tuz‐ Zohora

**Affiliations:** ^1^ Air Quality and Environmental Pollution Research Laboratory (AQEPRL), Centre for Advanced Research in Sciences (CARS) University of Dhaka Dhaka Bangladesh; ^2^ Department of Chemistry International University of Business Agriculture and Technology (IUBAT) Dhaka Bangladesh; ^3^ Department of Pharmacy University of Asia Pacific Dhaka Bangladesh

**Keywords:** breast cancer risk, diabetes, epidemiology, incidence, mortality, prognosis

## Abstract

**Introduction:**

Increasing epidemiological evidence suggests that diabetes is associated with a modest but significant elevation in breast cancer risk, as well as poorer clinical outcomes following diagnosis. This integrative review critically synthesizes current epidemiological, biological and clinical evidence to elucidate the role of diabetes in breast cancer development and progression among women.

**Methods:**

We conducted a structured literature search (2010–2025) to identify original studies examining the relationship between diabetes and breast cancer risk and outcomes. English‐language human studies were included. Titles, abstracts and full texts were screened against predefined criteria, and relevant data were extracted and synthesized narratively.

**Results:**

Studies indicate that women with diabetes experience an increased incidence of breast cancer, with stronger associations observed in postmenopausal populations, and partial attenuation of risk following adjustment for obesity, highlighting both shared and independent metabolic contributions. Mechanistically, chronic hyperinsulinemia, altered insulin‐like growth factor signalling, low‐grade systemic inflammation, oxidative stress and oestrogen‐metabolic crosstalk are frequently proposed as biologically plausible pathways through which diabetes may influence breast carcinogenesis, particularly in hormone‐receptor‐positive tumours. Clinically, diabetes has been linked to delayed diagnosis, increased treatment‐related complications, and higher breast cancer‐specific mortality, underscoring the need for integrated metabolic and oncologic care.

**Conclusion:**

Diabetes mellitus, particularly type 2 diabetes, is consistently associated in observational studies with a small increment in breast cancer risk and a more clearly adverse impact on survival. Mechanistic and translational evidence supports biologically plausible pathways linking metabolic dysregulation to tumour initiation, progression and treatment tolerance, but definitive causal effects remain uncertain. Medication and biomarker‐based research and practice should be incorporated in metabolic‐oncologic care and research to enhance prevention and outcomes.

## Graphical abstract

Type 2 diabetes mellitus is associated with a modest increase in breast cancer risk, particularly among postmenopausal women, with the association partially attenuated after adjustment for obesity. Hyperinsulinemia, IGF‐1 signalling, chronic inflammation and oestrogen dysregulation provide biologically plausible mechanisms linking diabetes to breast carcinogenesis. Clinically, diabetes is associated with delayed diagnosis, increased treatment‐related complications and higher mortality. Collectively, these findings support the need for integrated metabolic‐oncologic care to improve prevention, treatment and survivorship in women with diabetes and breast cancer.

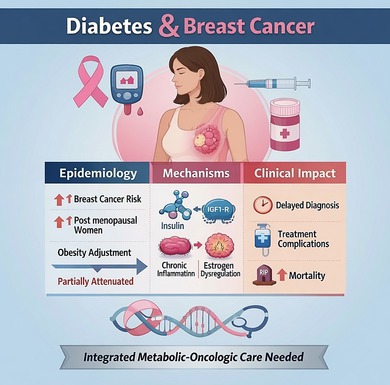

## 1 Introduction

Diabetes mellitus (DM) and breast cancer (BC) are two of the most prevalent and interlinked non‐communicable diseases affecting women globally. The prevalence of type 2 diabetes mellitus (T2DM) has risen dramatically over the past decades, largely due to lifestyle changes, urbanization and ageing populations. According to the International Diabetes Federation [1], more than 240 million women worldwide are currently living with diabetes, and the number is expected to rise steeply by 2045. Simultaneously, breast cancer remains the most frequently diagnosed cancer among women and is a leading cause of cancer‐related death [2].

Emerging epidemiological and clinical evidence suggests a bidirectional relationship between metabolic disorders such as diabetes and oncogenesis. In particular, the co‐occurrence of diabetes and breast cancer in women has been increasingly reported, raising important questions about shared biological mechanisms and potential causality. Epidemiological surveys have consistently shown the risk enhancement of breast cancer in patients with diabetes, with the risk elevation of about 20% reported over patients without diabetes [3, 4]. A meta‐analysis of random‐effects model supporting these findings also indicated a higher risk of breast cancer development by 27% among women living with type 2 diabetes [4].

Understanding the diabetes‐breast‐cancer relationship is crucial because both conditions are chronic, multifactorial diseases with overlapping risk determinants such as obesity, sedentary lifestyle, hormonal dysregulation and inflammation. Diabetes may plausibly influence both the initiation and progression of breast cancer through complex metabolic and hormonal pathways, although most supporting data in humans are observational. Hyperglycemia and hyperinsulinemia can promote cellular proliferation, impair apoptosis and enhance the bioavailability of insulin‐like growth factor‐1 (IGF‐1), which acts as a mitogen in breast tissue [5]. Furthermore, chronic inflammation associated with insulin resistance and adipocytokine imbalance (e.g., elevated IL‐6, TNF‐α, leptin) can create a microenvironment conducive to tumour initiation and progression [6].

From a clinical perspective, the coexistence of diabetes may adversely affect breast‐cancer outcomes. Studies have reported that women with diabetes are more likely to be diagnosed at advanced stages and have poorer overall and cancer‐specific survival [7, 8]. This could result from metabolic complications, reduced access to screening, and potential interference of anti‐diabetic or anti‐cancer medications. Moreover, the chronic inflammatory state and altered immune response seen in diabetes may reduce treatment efficacy or increase toxicity during chemotherapy and radiotherapy [9].

In low‐ and middle‐income countries, including Bangladesh, where the prevalence of both diabetes and breast cancer is steadily increasing, the intersection of these two diseases poses an emerging public‐health challenge. Late diagnosis, limited screening programmes and lack of integrated care models exacerbate the problem. Identifying women with diabetes as a high‐risk group for breast cancer may allow for targeted screening and prevention strategies, potentially improving early detection rates and outcomes [10, 11]. Although this review focuses on type 2 diabetes, many of the implicated pathways—including chronic low‐grade inflammation, oxidative stress and pro‐angiogenic signalling—are also prominent in other chronic conditions such as obesity, metabolic syndrome, non‐alcoholic fatty liver disease and cardiovascular disease [12, 13]. Understanding how these shared mechanisms modify breast‐cancer risk and outcomes could inform more integrated prevention and survivorship strategies across multiple comorbid conditions, rather than viewing diabetes as an isolated risk factor [12]. Therefore, this review aims to:

- Synthesize existing evidence on the relationship between diabetes and breast‐cancer development, progression and prognosis in women.
- Explore the biological mechanisms linking metabolic dysfunction in diabetes to oncogenic processes in breast tissue, including insulin/IGF signalling, inflammation, oxidative stress and hormonal modulation.
- Discuss clinical and therapeutic implications, including diagnostic challenges, treatment responses and management strategies for women with both conditions.
- Identify controversies, knowledge gaps, and future directions for research to guide precision‐medicine and public‐health interventions.
- Provide an integrated understanding of epidemiological data, mechanistic insights, and clinical implications that highlight the importance of metabolic health in breast‐cancer prevention and management.

## 2 Methods for Integrative Review

### 2.1 Study Design and Review Approach

This study employed an integrative review design to comprehensively synthesize epidemiological, biological and clinical evidence examining the relationship between diabetes mellitus and breast cancer development, progression and outcomes in women. The integrative review methodology was selected because it permits the inclusion and critical interpretation of diverse study designs, including observational epidemiology, mechanistic research and clinical outcome studies, thereby enabling a holistic understanding of complex metabolic‐oncologic interactions. The methodological framework was informed by established integrative review principles and followed the core transparency and reporting concepts of the PRISMA guidelines, adapted appropriately for narrative and thematic synthesis rather than quantitative meta‐analysis. Although ‘diabetes mellitus’ encompasses heterogeneous entities, the epidemiological and mechanistic literature synthesized in this review predominantly concerns type 2 diabetes mellitus (T2DM); therefore, our conclusions primarily apply to T2DM in adult women unless otherwise specified.

### 2.2 Literature Search Strategy and Data Sources

**FIGURE 1:**
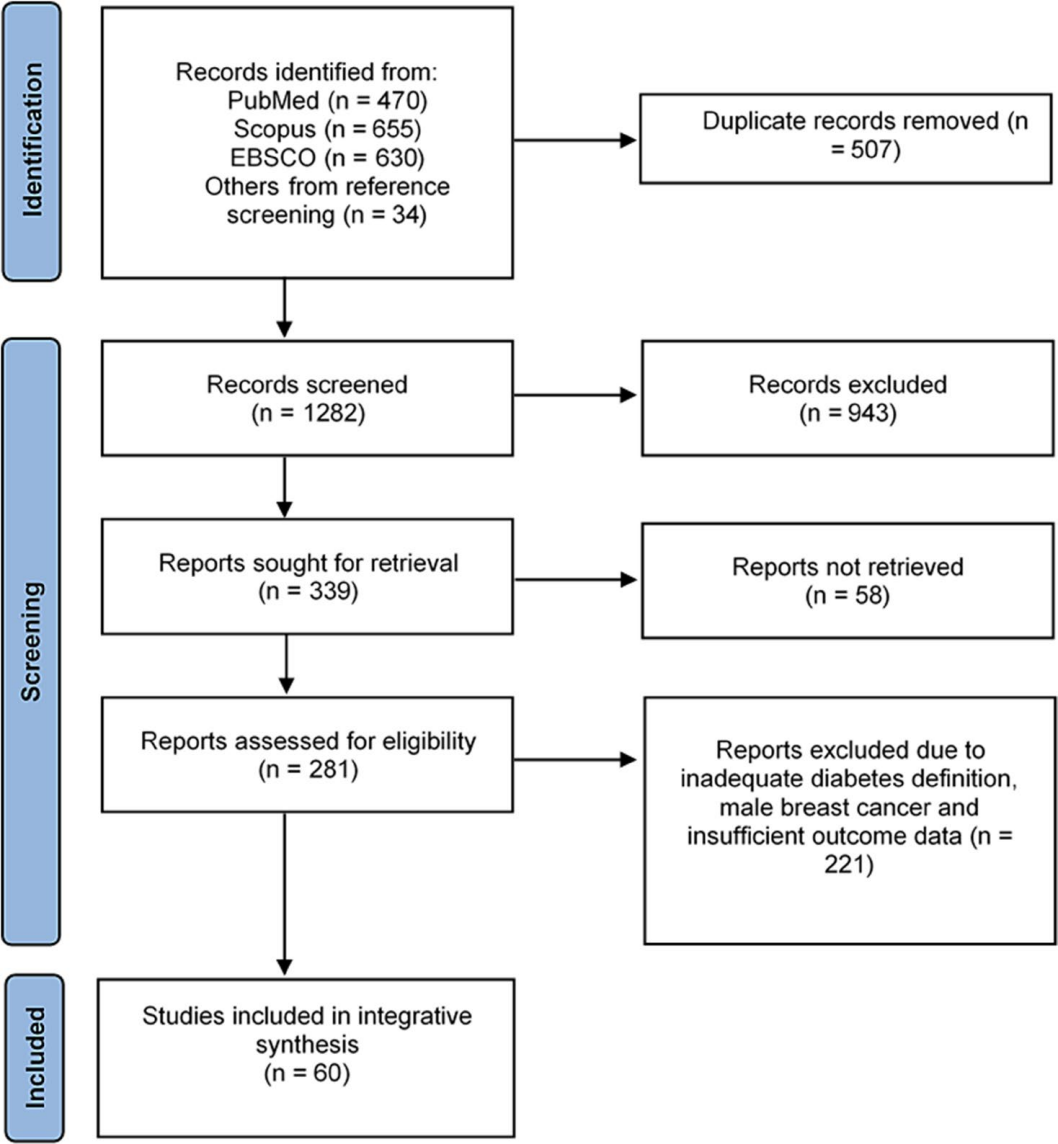
PRISMA‐adapted flow diagram to illustrate the study identification and selection process for this integrative review.

A systematic and comprehensive literature search was conducted across multiple electronic databases, including PubMed, Scopus and EBSCO, to identify relevant peer‐reviewed publications. The search strategy combined controlled vocabulary terms and free‐text keywords related to diabetes mellitus and breast cancer, encompassing epidemiological risk, prognosis, biological mechanisms and clinical implications. For example, a core PubMed search string was: (‘Diabetes Mellitus, Type 2’ [Majr] OR ‘type 2 diabetes’ [tiab] OR T2DM [tiab]) AND (‘Breast Neoplasms’ [Majr] OR ‘breast cancer’ [tiab] OR ‘breast neoplasm*’ [tiab]) AND (risk [tiab] OR incidence [tiab] OR prognosis [tiab] OR survival [tiab] OR mortality [tiab] OR outcome* [tiab]) AND (‘2010/01/01’ [dp]: ‘2025/12/31’ [dp]) AND Humans [Mesh] AND Female [Mesh]. This strategy was adapted for Scopus, and EMBASE using database‐specific subject headings and syntax, and the references of included studies were screened to capture additional recent literature. Boolean operators were used to refine search sensitivity and specificity. The search covered studies published between 1 January 2010 and 31 December 2025 and was limited to human studies in adult women and articles written in English. Across all databases, the search retrieved a total of 1789 records; after removal of 507 duplicates, 1282 unique titles and abstracts were screened. Of these, 281 full‐text articles were assessed for eligibility, and 60 studies meeting the predefined criteria were included in the final synthesis, as depicted in the PRISMA‐style flow diagram (Figure 1).

### 2.3 Eligibility Criteria and Study Selection

Studies were selected based on predefined inclusion criteria to ensure relevance and methodological appropriateness. Eligible publications included peer‐reviewed articles written in English that involved human female populations and examined type 2 diabetes mellitus in relation to breast cancer incidence, progression, prognosis, survival, biological mechanisms or therapeutic implications. Observational studies, clinical investigations, translational research, systematic reviews and meta‐analyses were included. Articles focusing exclusively on non‐breast malignancies, male breast cancer, animal or in vitro models without clinical relevance, case reports, editorials and conference abstracts were excluded. Titles and abstracts were screened for relevance, followed by full‐text evaluation to confirm eligibility, with inclusion decisions made through iterative review and consensus among the authors.

### 2.4 Data Extraction and Evidence Synthesis

Relevant data were systematically extracted from included studies, capturing information on study design, population characteristics, diabetes definition and duration where available, breast cancer outcomes, effect estimates, mechanistic pathways and clinical implications. Given the substantial heterogeneity in exposure definitions, outcome measures, analytical adjustments, and study designs, quantitative pooling or meta‐analysis was not undertaken. Instead, findings were synthesized using a narrative and thematic approach. Evidence was organized conceptually across epidemiological associations, biological and pathophysiological mechanisms, and clinical implications, allowing integration of population‐level data with mechanistic plausibility and real‐world clinical relevance.

### 2.5 Quality Considerations, Bias Assessment and Reporting Transparency

During interpretation, methodological quality and potential sources of bias were critically considered, including confounding by obesity and lifestyle factors, screening and detection bias, heterogeneity by menopausal status and tumour subtype, and confounding by indication in medication‐based studies. Particular attention was paid to clarity of diabetes definition, ascertainment of breast‐cancer outcomes, adjustment for key confounders (e.g., age, BMI, menopausal status), sample size, and follow‐up duration when weighing the contribution of individual studies to the overall narrative. Rather than excluding studies based solely on methodological variation, these limitations were explicitly acknowledged and incorporated into the interpretive framework to contextualize findings.

## 3 Epidemiological Evidence Linking Diabetes and Breast Cancer

### 3.1 Diabetes as a Risk Factor for Breast‐Cancer Incidence

A substantial body of epidemiological research has examined whether diabetes increases the risk of breast cancer among women. Multiple prospective cohort studies have evaluated incident breast‐cancer risk among women with diabetes. Recent evidence from a large systematic review and meta‐analysis indicates that women with diabetes have a modestly increased breast cancer risk overall (RR ≈ 1.20) and that the association remains significant in postmenopausal women (RR ≈ 1.12), suggesting differential effects by menopausal status and tumour subtype [13]. Meta‐analytic evidence further supports this association. A comprehensive review reported that women with diabetes experienced approximately 27% higher breast‐cancer risk before BMI adjustment; however, when obesity was included in statistical models, the association was attenuated to around 12% [4]. Most of the contributing cohorts and meta‐analyses predominantly include women with type 2 diabetes, and only a minority of studies distinguish clearly between diabetes types. Accordingly, the modest risk elevation observed is best interpreted as reflecting the typical metabolic profile of T2DM in adult women rather than all forms of diabetes.

This attenuation pattern suggests that diabetes and obesity share overlapping biological determinants, including hyperinsulinemia, chronic inflammation and oestrogen overproduction through aromatase activity in adipose tissue [9]. Importantly, available evidence indicates that risk differences may vary according to menopausal status, with stronger associations observed in postmenopausal women, possibly due to endocrine changes associated with insulin resistance [14].

### 3.2 Impact of Diabetes on Breast‐Cancer Prognosis and Outcomes

Beyond incidence, epidemiological studies consistently demonstrate that diabetes negatively influences breast‐cancer prognosis and survival outcomes. Women with diabetes are more likely to present with advanced‐stage disease and experience higher all‐cause and cancer‐specific mortality compared with non‐diabetic breast‐cancer patients [15]. Where reported, adverse prognostic effects tend to be more pronounced among women with longer‐standing diabetes, poor glycemic control, and coexisting obesity, but such patterns remain heterogeneous across studies and are difficult to interpret because of confounding by comorbidity burden and treatment intensity [16].

Peairs et al. [17] first showed that pre‐existing diabetes was associated with higher mortality in breast cancer patients. More recent evidence confirms that diabetes mellitus adversely affects survival outcomes, with narrative epidemiologic reviews reporting that diabetes is associated with increased all‐cause and cancer‐specific mortality among breast cancer patients [18]. Similar trends were observed in later studies, which documented increased recurrence risk, greater treatment‐related complications and reduced survival among women with diabetes [7]. Potential explanations include biological effects of metabolic dysfunction, under‐treatment due to comorbid conditions, delayed diagnosis and differences in treatment tolerance.

### 3.3 Role of Confounding, Bias and Effect Modification

Despite the consistent relationship between diabetes and breast‐cancer risk and outcome, residual confounding and bias make it difficult to interpret epidemiologically. Similar risks like obesity, physical inactivity and dietary habits might partially explain the observed associations, whereas screening‐related bias and late presentation in women with diabetes will also affect the stage at diagnosis and survival rates. Furthermore, heterogeneity exists due to effect modification by body mass index, menopausal status, ethnicity and antidiabetic medication use, which highlights the importance of caution when interpreting the results and conducting properly planned prospective studies [19].

### 3.4 Clinical Interpretation of Epidemiological Evidence

In general, epidemiological research shows that diabetes mellitus correlates with a small rise in the risk of breast cancer and a steadier negative effect on prognosis and survival. These associations have been documented most consistently in women with type 2 diabetes, and are partially attenuated but not always eliminated after adjustment for obesity and related lifestyle factors. Within T2DM populations, longer diabetes duration, poorer glycemic control (higher HbA1c), coexisting obesity or metabolic syndrome, and use of exogenous insulin often cluster and appear to amplify risk and worsen breast‐cancer outcomes, although few studies provide sufficiently detailed stratification to disentangle their individual effects.

These findings, in the clinical sense, can serve in supporting the significance of early detection of breast‐cancer in women with diabetes, the combination of metabolic and oncologic care, and further research efforts that should include refined risk‐stratification and precision‐prevention initiatives [9].

Key epidemiological studies and meta‐analyses examining the association between diabetes and breast cancer risk are summarized in Table 1.

**TABLE 1:** Epidemiological evidence linking diabetes and breast cancer risk in women.

Study	Study type	Population	Key findings	Notes
Xiong et al. [13]	Systematic review and meta‐analysis	> 70 cohort and case–control studies	Diabetes associated with increased breast cancer risk (RR ≈ 1.20); stronger association in postmenopausal women	Subtype‐specific variation reported
Zhou et al. [20]	Systematic review and meta‐analysis (with in silico analysis)	20 studies; > 2.6 million participants, including > 207,000 women with diabetes	Pre‐existing diabetes was associated with increased all‐cause mortality (HR = 1.37, 95% CI 1.34–1.41) and breast‐cancer‐specific mortality (HR = 1.17, 95% CI 1.11–1.22) in women with breast cancer	Suggests adverse prognostic impact of diabetes; exploratory in silico analysis indicated potential antitumor effects of metformin via microRNA modulation
Jordt et al. [21]	Systematic review and meta‐analysis	15 observational studies; > 80,000 breast cancer survivors	Breast cancer survivors had an increased risk of incident type 2 diabetes compared with non‐cancer controls (pooled EE = 1.23, 95% CI 1.13–1.33); tamoxifen therapy further increased diabetes risk (EE = 1.28, 95% CI 1.18–1.38)	Highlights bidirectional relationship; treatment‐related metabolic effects may contribute to diabetes development
Zhao & Ren [8]	Systematic review and meta‐analysis	17 cohort and case–control studies; 48,315 women with breast cancer	Pre‐existing diabetes was independently associated with worse overall survival (HR = 1.51, 95% CI 1.34–1.70) and disease‐free survival (HR = 1.28, 95% CI 1.09–1.50); no significant association with relapse‐free period	Consistent adverse prognostic effect across regions and study designs; heterogeneity partly related to diabetes definition and follow‐up duration
Peairs et al. [17]	Systematic review and meta‐analysis	8 observational studies; > 48,000 women with breast cancer	Pre‐existing diabetes was associated with significantly higher all‐cause mortality (pooled HR = 1.49, 95% CI 1.35–1.65); several studies also reported more advanced stage at diagnosis and altered treatment patterns in patients with diabetes	Robust association across study designs; heterogeneity present; findings independent of major confounders; highlights impact of comorbidity on cancer outcomes
Bhatia et al. [22]	Systematic review and meta‐analysis	37 observational studies; adult women with and without diabetes	Women with diabetes were significantly less likely to undergo breast cancer screening compared with women without diabetes (pooled OR = 0.83, 95% CI 0.77–0.90), indicating lower mammography uptake	Screening disparities may contribute to delayed breast cancer diagnosis and poorer outcomes in women with diabetes
Li et al. [23]	Systematic review & meta‐analysis	19 cohort and case–control studies; > 3 million women	No significant overall association between gestational diabetes mellitus (GDM) and breast cancer risk (pooled HR = 1.03, 95% CI 0.92–1.15); substantial regional heterogeneity observed	GDM associated with reduced risk in North America (HR = 0.89) but increased risk in Asian populations (HR = 1.23), highlighting geographic and metabolic‐context effects
Michels et al. [24]	Prospective cohort	Nurses' Health Study	Elevated risk in women with diabetes	Historical evidence
Zhang et al. [25]	Systematic review and meta‐analysis	17 observational studies; women with breast cancer and pre‐existing T2DM	T2DM was associated with advanced tumour stage (III/IV vs. in situ–II: pooled OR = 1.19, 95% CI 1.04–1.36), larger tumour size (> 20 mm vs. ≤ 20 mm: OR = 1.18, 95% CI 1.04–1.35), and lymph node invasion (OR = 1.26, 95% CI 1.05–1.51)	Indicates more aggressive disease at diagnosis in women with T2DM; findings support a role for delayed detection and diabetes‐related metabolic effects
Simon et al. [26]	Systematic review	14 observational studies (10 cohort, 4 case–control); women with prior gestational diabetes mellitus	Evidence was inconsistent: 3 studies reported increased breast cancer risk after GDM, 3 reported decreased risk, and 8 found no significant association; pooled meta‐analytic evidence from prior studies showed no significant overall association	Highlights substantial heterogeneity by region, age at GDM, menopausal status, and study quality; conclusions limited by exposure misclassification and residual confounding

*Note:* Reported estimates reflect pooled or adjusted measures as presented in the respective studies; heterogeneity and covariate adjustment varied across analyses.

Abbreviations: CI, confidence interval; EE, effect estimate; GDM, gestational diabetes mellitus; HR, hazard ratio; OR, odds ratio; RR, relative risk; T2DM, type 2 diabetes mellitus.

## 4 Biological and Pathophysiological Mechanisms Linking Diabetes and Breast Cancer

### 4.1 Key Metabolic Features of Type 2 Diabetes Relevant to Cancer

Diabetes mellitus is a heterogeneous metabolic disorder characterized by chronic hyperglycemia resulting from impaired insulin secretion, insulin resistance, or a combination of both. Type 2 diabetes mellitus (T2DM), which constitutes the majority of global diabetes cases among women, develops gradually through progressive β‐cell dysfunction superimposed on peripheral insulin resistance. These abnormalities lead to altered glucose uptake in skeletal muscle, increased hepatic glucose production and dysregulated lipid metabolism [27].

The metabolic environment associated with T2DM extends beyond hyperglycemia. Individuals often present with central obesity, dyslipidemia, systemic inflammation, and elevated free fatty acids‐elements collectively associated with metabolic syndrome. This metabolic background promotes chronic hyperinsulinemia as pancreatic β‐cells initially attempt to compensate for reduced insulin sensitivity. Over time, persistent metabolic stress contributes to oxidative stress, mitochondrial dysfunction and inflammatory cytokine activation [28].

From an endocrine perspective, insulin is not only a metabolic hormone but also possesses mitogenic potential. Insulin receptors and hybrid insulin/IGF receptors are expressed in many tissues, including breast epithelium. In the diabetic milieu, sustained exposure to elevated insulin and IGF‐1 signalling may favour cellular proliferation and impair programmed cell death. These alterations are widely cited as a key hypothesized mechanistic bridge between diabetes and cancer, particularly breast cancer, where hormonal and metabolic pathways overlap [9].

### 4.2 Breast Cancer Biology Relevant to Metabolic Dysregulation

Breast cancer is a complex and biologically diverse malignancy arising from genetic, hormonal and environmental interactions. Tumour development typically involves multi‐step genetic alterations affecting cell‐cycle regulation, DNA repair, apoptosis and growth signalling pathways. Molecular subtyping, including hormone receptor‐positive (ER/PR+), HER2‐enriched, and triple‐negative breast cancer, has enhanced understanding of tumour behaviour and therapeutic responsiveness [29].

Endocrine regulation plays a central role in breast‐cancer biology. Oestrogen promotes proliferation of breast epithelial cells through receptor‐mediated transcriptional activation, increasing the likelihood of DNA replication errors and genetic instability. Obesity and insulin resistance, frequently observed in women with diabetes, further amplify oestrogen exposure through increased aromatase activity in adipose tissue, leading to higher circulating oestrogen levels in postmenopausal women [30].

In addition to hormonal pathways, metabolic signalling networks such as PI3K/Akt/mTOR, MAPK and IGF pathways contribute to tumour growth and survival. Many of these pathways are shared with metabolic disease mechanisms. This convergence is an important theoretical basis for understanding why diabetes may influence not only breast‐cancer risk but also tumour aggressiveness and response to therapy [9].

### 4.3 Mechanistic Links Between Diabetes and Breast‐Cancer Development

**FIGURE 2:**
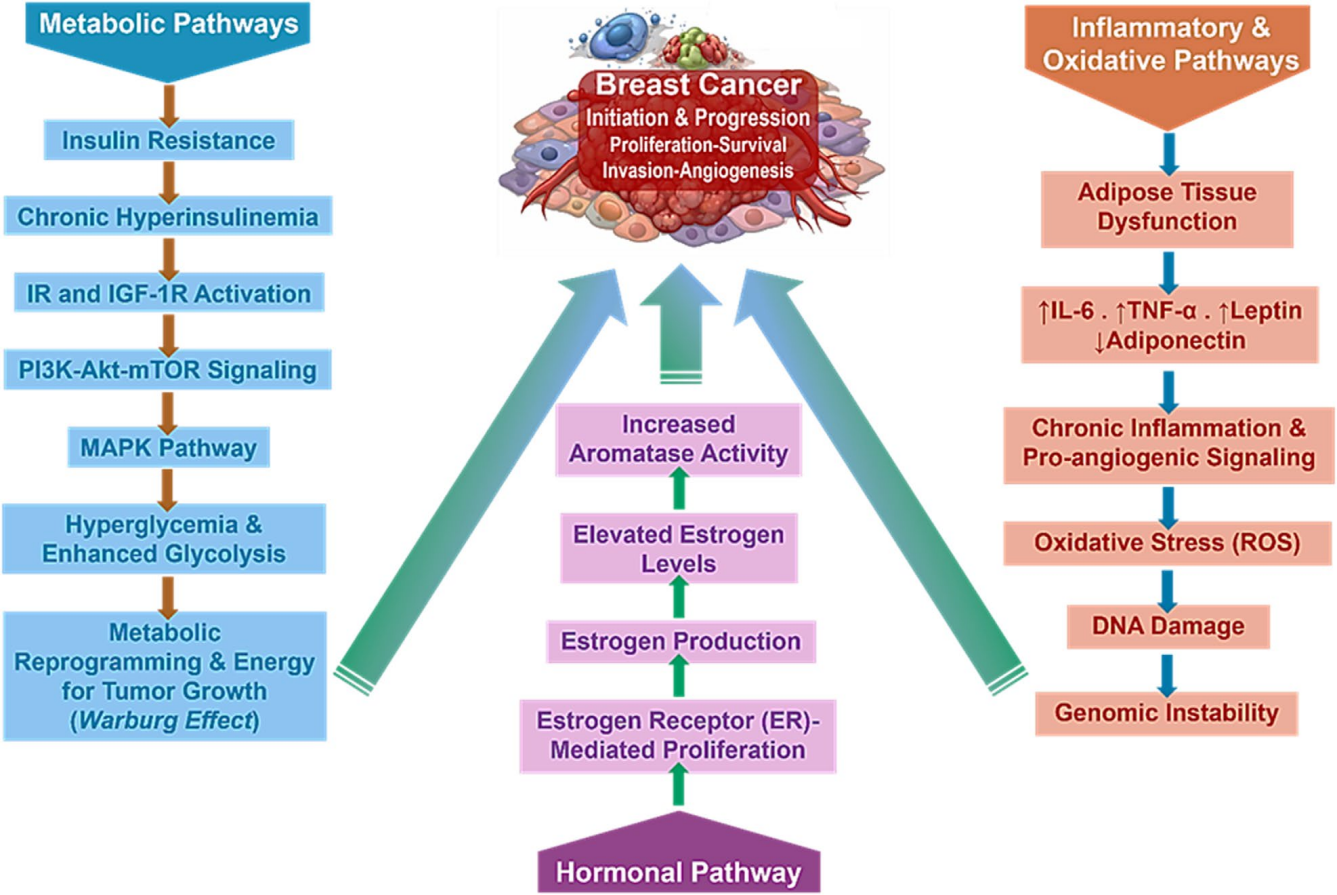
Integrated biological pathways linking type 2 diabetes mellitus to breast cancer development and progression. This schematic figure illustrates the interconnected metabolic, inflammatory/oxidative and hormonal mechanisms through which type 2 diabetes mellitus (T2DM) contributes to breast cancer initiation and progression. The metabolic axis involves insulin resistance‐induced hyperinsulinemia, activation of insulin receptor (IR) and insulin‐like growth factor‐1 receptor (IGF‐1R), and downstream PI3K‐Akt–mTOR and MAPK signalling pathways, which promote mitogenic and survival signalling. Hyperglycemia further enhances glycolysis and metabolic reprogramming (Warburg effect), providing bioenergetic and biosynthetic support for tumour growth. The inflammatory and oxidative axis is driven by adipose tissue dysfunction associated with obesity and T2DM, characterised by increased IL‐6, TNF‐α, and leptin and reduced adiponectin, leading to chronic inflammation, pro‐angiogenic signalling, oxidative stress (ROS), DNA damage and genomic instability. The hormonal axis highlights increased aromatase activity, elevated oestrogen production, and oestrogen receptor (ER)‐mediated transcription of proliferative genes, particularly relevant in postmenopausal women. Collectively, these converging pathways foster tumour proliferation, survival, invasion, angiogenesis and microenvironmental remodelling.

A growing body of literature proposes multiple interconnected mechanisms that may help explain observed associations between diabetes and breast‐cancer initiation and progression. Rather than a single causal pathway, the relationship is best conceptualized as a network of metabolic, hormonal, inflammatory and molecular interactions. The mechanistic convergence of insulin signalling, chronic inflammation, oxidative stress and oestrogen receptor activation in T2DM‐driven breast cancer is summarized in Figure 2.

#### 4.3.1 Hyperinsulinemia and IGF‐1 Signalling

Women with T2DM frequently experience chronic elevations in circulating insulin and, in some cases, increased IGF‐1 bioactivity. Insulin and IGF‐1 can activate mitogenic signalling cascades in breast tissue, including PI3K/Akt and Ras/MAPK pathways, which promote proliferation, inhibit apoptosis and facilitate tumour cell survival [31]. Changes in IGF‐binding proteins, particularly reductions in IGFBP‐3, may increase the bioavailability of free IGF‐1, amplifying these growth‐promoting effects [32]. Experimental and epidemiological studies suggest that this mechanism may be especially relevant in hormone‐receptor‐positive tumours, where growth‐factor signalling interacts with oestrogen‐receptor activity [33].

#### 4.3.2 Chronic Inflammation and Adipocytokine Dysregulation

Diabetes and obesity are characterized by low‐grade, systemic inflammation. Adipose tissue secretes pro‐inflammatory cytokines such as IL‐6, TNF‐α and leptin, while adiponectin, a metabolically protective hormone, is typically reduced. These inflammatory mediators contribute to oxidative stress, stimulate angiogenesis and support tumour‐promoting microenvironmental changes [34]. This inflammatory state may accelerate tumour initiation and progression by facilitating DNA damage and impairing immune surveillance. The convergence of inflammatory and metabolic stress therefore creates conditions favourable for carcinogenesis [35]. Beyond their role in diabetes‐associated metabolic dysfunction, chronic inflammatory processes may represent a broader biological framework linking several chronic diseases to breast cancer development and progression. Persistent activation of inflammatory mediators such as IL‐6, TNF‐α and NF‐κB signalling can promote angiogenesis, genomic instability, immune dysregulation and tumour microenvironment remodelling. In particular, pro‐angiogenic signalling pathways driven by chronic inflammation may contribute to tumour growth, invasion and metastatic potential [12, 36].

#### 4.3.3 Hyperglycemia and Cellular Energy Metabolism

Persistent hyperglycemia provides an abundant substrate supply for rapidly dividing tumour cells. Cancer cells preferentially utilize glycolytic metabolism, a phenomenon often referred to as the ‘Warburg effect’. Elevated glucose availability may enhance glycolytic flux and support biosynthetic pathways necessary for tumour growth [37].

#### 4.3.4 Hormonal Interactions and Oestrogen Signalling

Insulin resistance and obesity frequently alter sex‐hormone metabolism. Increased aromatase activity in adipose tissue elevates oestrogen levels, particularly after menopause. Higher oestrogen exposure synergizes with insulin and IGF‐1 signalling, augmenting proliferative signalling in oestrogen‐dependent breast tissue [30].

#### 4.3.5 Medication‐Related Pathways (Metformin vs. Insulin Therapy)

Pharmacologic treatment of diabetes may also modify cancer risk and outcomes. Observational research suggests that metformin may have anti‐tumour properties through AMPK activation and inhibition of mTOR signalling [38, 39], whereas exogenous insulin could theoretically enhance mitogenic signalling pathways [31].

#### 4.3.6 Adipose‐Derived Factors and Tumour‐Microenvironment Interactions

Adipose tissue in diabetic individuals undergoes structural remodelling characterized by macrophage infiltration, fibrosis and hypoxia. This environment favours secretion of leptin, resistin and pro‐tumorigenic lipids, which promote cell proliferation, angiogenesis and epithelial‐mesenchymal transition. Reduced adiponectin removes an important inhibitory signal on tumour growth and insulin signalling [40].

Taken together, the theoretical framework suggests that diabetes and breast cancer share overlapping biological pathways involving metabolic dysregulation, growth‐factor signalling, inflammation and hormonal regulation. Rather than viewing diabetes merely as a coincidental comorbidity, it may be considered a plausible biological modifier that could influence cancer development, phenotype, and clinical trajectory, although current evidence is insufficient to confirm a direct causal effect. This conceptual understanding provides the foundation for examining epidemiological patterns, prognostic associations and therapeutic interactions in subsequent sections of the review.

It is important to emphasize that these mechanistic pathways provide a coherent biological framework but do not in themselves prove that diabetes causes breast cancer. The epidemiological data summarized in this review primarily demonstrate statistical associations, which may reflect a combination of shared risk factors, mediation by metabolic traits and potentially some independent effects of diabetes‐related dysregulation.

## 5 Clinical Implications

This section highlights clinical implications that are supported by current observational evidence, established diabetes and oncology guidelines, or widely accepted expert consensus. Speculative strategies and research priorities are discussed separately in the ‘Future Directions’ section to distinguish evidence‐based practice from areas where further study is required.

### 5.1 Clinical Relevance of Diabetes in Breast Cancer

**FIGURE 3:**
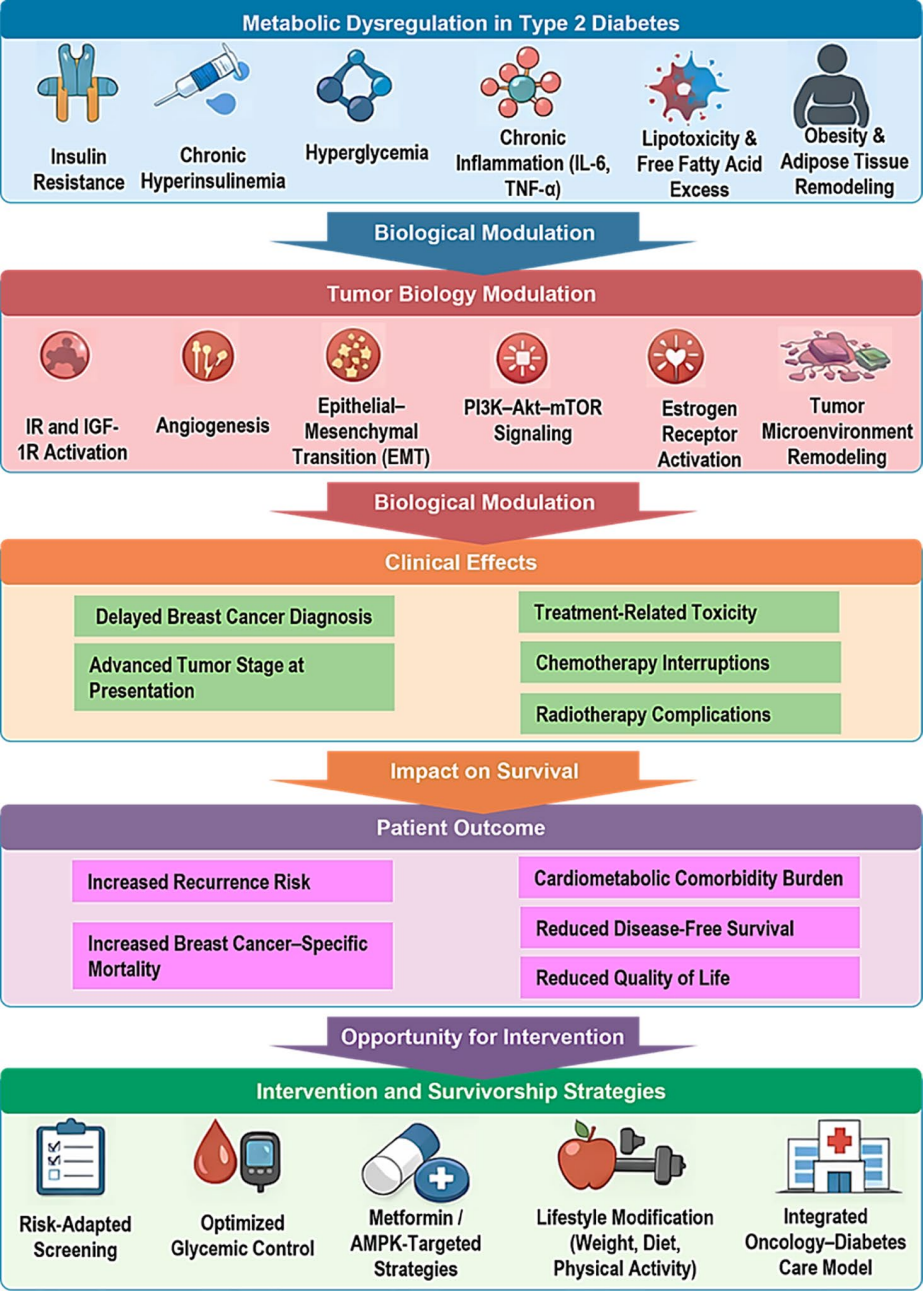
Translational framework illustrating the continuum from metabolic dysregulation in type 2 diabetes to breast cancer outcomes and survivorship. The illustration shows metabolic disturbances in type 2 diabetes mellitus (T2DM)–including insulin resistance, hyperinsulinemia, hyperglycemia, lipotoxicity, chronic inflammation and obesity‐related adipose remodelling–create a pro‐tumorigenic environment. These alterations modulate tumour biology through IR/IGF‐1R activation, PI3K‐Akt–mTOR signalling, oestrogen receptor stimulation, angiogenesis, epithelial‐mesenchymal transition and microenvironment remodelling. Clinically, these effects are associated with delayed diagnosis, advanced tumour stage, treatment‐related complications, and therapy interruptions, contributing to increased recurrence risk, reduced survival and greater cardiometabolic burden. The framework also highlights intervention opportunities, including risk‐adapted screening, optimized glycemic control, metabolic‐targeted therapies, lifestyle modification and integrated oncology‐diabetes care.

The coexistence of diabetes and breast cancer has important clinical implications that extend beyond epidemiological associations. Diabetes may influence multiple stages of the cancer continuum, from diagnosis and treatment selection to prognosis, survivorship and quality of life. The metabolic derangements associated with diabetes, together with treatment‐related interactions and comorbidity burden, can complicate clinical decision‐making and may contribute to suboptimal outcomes if not adequately addressed in routine care. Understanding these implications is essential for oncologists, endocrinologists, primary‐care physicians and public‐health professionals, particularly in settings where the prevalence of both diabetes and breast cancer is rising [9]. To summarize these multi‐level clinical consequences and intervention opportunities across the cancer continuum, a translational framework is presented in Figure 3. The concept of integrated metabolic‐oncologic care is thus grounded in existing evidence on comorbidity burden and treatment complications and is increasingly reflected in expert recommendations for multidisciplinary management, even though specific models of care and their impact on hard outcomes remain under active investigation.

### 5.2 Implications for Screening and Early Detection

Several studies report that women with diabetes are more likely to be diagnosed with breast cancer at later stages, which adversely affects survival. Potential contributors include competing health priorities, reduced health‐seeking behaviour, limited awareness and underutilization of screening programmes. Some authors have proposed that women with diabetes may benefit from enhanced breast‐cancer surveillance, particularly if additional risk factors such as obesity, postmenopausal status or poor glycemic control are present. Although formal international guidelines do not currently designate diabetes as a standalone indication for intensified screening, clinicians are encouraged to apply a risk‐adapted approach, ensuring adherence to mammography schedules and addressing barriers to participation [7, 41]. These suggestions are based on observational evidence and expert opinion rather than randomized screening trials specifically targeting women with diabetes, and current recommendations focus on ensuring adherence to standard age‐ and risk‐based mammography schedules rather than introducing diabetes‐specific screening protocols.

### 5.3 Influence of Diabetes on Treatment Decision‐Making and Outcomes

Diabetes may affect the tolerance, safety and effectiveness of breast‐cancer therapies. Observational studies have reported higher rates of postoperative complications, chemotherapy toxicity and treatment interruptions among women with diabetes [42]. Hyperglycemia and insulin resistance can impair wound healing, increase infection risk and alter pharmacokinetics of cytotoxic agents. Chemotherapy‐induced metabolic disturbances, such as steroid‐associated hyperglycemia, may worsen glycemic control, creating a bidirectional management challenge [31].

Radiotherapy tolerance may also be affected by vascular and inflammatory changes associated with diabetes, while endocrine therapies may interact with metabolic pathways, particularly in oestrogen‐receptor‐positive disease. These complexities highlight the importance of multidisciplinary care, integrating oncology with diabetes management to optimize therapeutic delivery, consistent with current diabetes and cancer‐care guidelines and expert consensus even though direct trial evidence in breast‐cancer populations remains limited [19].

### 5.4 Glycemic and Metabolic Management During Cancer Therapy

Emerging evidence suggests that glycemic control may play a role in shaping breast‐cancer outcomes. Poorly controlled diabetes has been associated with higher mortality, increased recurrence risk and longer hospital stays [41]. Observational data suggest dose–response relationships in which higher HbA1c levels and longer diabetes duration are associated with worse breast‐cancer outcomes, although these gradients are not uniformly observed and may partly reflect greater comorbidity in patients with more severe diabetes.

Although randomized evidence remains limited, improving glycemic control during cancer treatment is considered clinically prudent and may support better tolerance of therapy, reduced complications and improved quality of life [19, 31, 35]. Multimodal strategies, including lifestyle modification, pharmacologic optimization and close glucose monitoring, are therefore recommended as part of comprehensive care, in line with current diabetes‐management guidelines and expert consensus, although high‐quality randomized data on breast‐cancer‐specific outcomes remain scarce [19].

### 5.5 Antidiabetic Medications and Breast Cancer Outcomes

Antidiabetic medications may differentially influence cancer outcomes. Metformin, widely discussed in the oncology literature, has been associated with favourable prognostic signals in some observational studies, potentially through AMPK activation and suppression of mTOR signalling [43]. Conversely, the use of exogenous insulin and certain insulin analogues has raised theoretical concerns due to their mitogenic potential, although findings remain inconsistent. Much of the current evidence is observational and confounded by disease severity and indication bias, as patients treated with insulin or complex regimens typically have longer disease duration and poorer glycemic control than those managed with lifestyle measures or metformin alone. Ongoing and future clinical trials are required to clarify whether diabetes medications exert direct anticancer or protumor effects, or whether observed differences primarily reflect underlying metabolic status [9]. At present, these observations should inform individualized clinical judgement and shared decision‐making rather than routine modification of standard breast‐cancer therapy based solely on diabetes medication class, pending more definitive trial data.

### 5.6 Comorbidity Burden and Health‐System Challenges

Women living with both diabetes and breast cancer often experience a higher overall comorbidity burden, which can influence treatment eligibility, recovery and survivorship outcomes. Polypharmacy, cardiovascular risk, neuropathy and renal impairment may complicate therapy choices and monitoring requirements. These challenges may be especially pronounced in resource‐constrained settings, where fragmented care and limited specialist coordination hinder optimal management [8, 17, 22].

### 5.7 Survivorship, Lifestyle Interventions and Long‐Term Care

After completion of active treatment, survivorship care represents a critical phase for women with diabetes. Weight management, physical activity and dietary interventions not only improve metabolic health but may also reduce recurrence risk and improve functional outcomes [44]. Lifestyle modification is therefore considered a cornerstone of long‐term management for breast‐cancer survivors with diabetes. In addition to improving glycemic control, lifestyle interventions that promote weight loss, increase physical activity and favour anti‐inflammatory dietary patterns, such as Mediterranean‐style diets, may help lower chronic inflammatory and oxidative stress burden, which are hypothesized contributors to breast‐cancer progression in women with diabetes [27, 44]. Emerging evidence suggests that such interventions can favourably modify circulating inflammatory and oxidative biomarkers, supporting their potential role as pragmatic survivorship strategies while more specific biomarker‐guided approaches are being developed [45, 46].

Psychosocial factors such as stress, fatigue and reduced physical capacity may further complicate diabetes self‐management during survivorship. Coordinated follow‐up that includes metabolic monitoring, cardiovascular risk assessment and behavioural support can help sustain long‐term health and quality of life [27, 47].

### 5.8 Clinical Take‐Home Messages

Overall, the clinical evidence indicates that diabetes adversely affects several aspects of breast‐cancer care, including diagnosis, treatment tolerance and survival, largely through the combined effects of metabolic dysfunction, comorbidity burden and health‐system factors. Insights synthesized from supporting literature reinforce the importance of proactive screening, optimized glycemic management and multidisciplinary care models tailored to this high‐risk patient population. These implications form a bridge between mechanistic understanding and real‐world practice and provide a foundation for exploring ongoing debates and research gaps in the next section of the review [9].

## 6 Controversies and Debates

### 6.1 The Role of Obesity and Metabolic Factors as Confounders

One of the central debates concerns the relative contribution of obesity to the observed association between diabetes and breast cancer. Many studies report that risk estimates for breast cancer decline or lose statistical significance after adjusting for BMI. This pattern suggests that part of the relationship may be driven by adiposity rather than diabetes itself [4, 13, 48]. However, complete attribution of risk to obesity alone may oversimplify the relationship. Some studies demonstrate persistent risk elevation even after multivariable adjustment, implying that diabetes‐specific metabolic alterations (such as hyperinsulinemia, glycemic variability, or medication exposure) may independently influence cancer risk. Distinguishing these overlapping effects remains methodologically difficult, particularly in observational research [13, 19].

### 6.2 Causality vs. Association: Epidemiological Interpretation

Another controversy centres on whether current evidence supports causality or merely statistical association. Most studies examining diabetes and breast cancer rely on observational cohort or case–control designs, which are inherently vulnerable to residual confounding and reverse causation. For example, undiagnosed preclinical cancer may alter metabolic pathways before diagnosis, potentially influencing diabetes detection or management. Few studies incorporate advanced causal‐inference frameworks, biomarkers of insulin exposure, or longitudinal metabolic profiling [14, 24]. Without such approaches, assigning a direct causal pathway remains tentative. Mendelian‐randomization studies have produced mixed findings, further illustrating uncertainty about whether diabetes itself, or correlated biological traits, constitute the primary driver of risk [49]. Taken together, these findings support diabetes as an important marker and potential mediator of worse breast‐cancer risk and prognosis, but they fall short of establishing a direct, unconfounded causal relationship.

### 6.3 Screening Bias, Diagnostic Delay and Health‐System Effects

A further source of debate involves the possibility of screening and detection bias. Some evidence suggests that women with chronic illnesses, including diabetes, may undergo less frequent cancer screening or delay seeking evaluation for breast symptoms due to competing health concerns. This may contribute to the higher proportion of late‐stage diagnoses reported among women with diabetes. In such cases, worse outcomes may reflect health‐system inequities rather than intrinsic tumour biology [50]. Conversely, frequent healthcare contact for diabetes management could theoretically increase opportunities for cancer detection, leading to earlier diagnosis in some contexts. These counteracting forces make interpretation of stage‐at‐diagnosis differences complex [51, 52].

### 6.4 Heterogeneity by Tumour Subtype and Menopausal Status

Another debated area concerns whether diabetes exerts different effects across breast‐cancer subtypes and menopausal groups. Evidence suggests that associations may be stronger for oestrogen‐receptor‐positive tumours, reflecting interaction between metabolic signalling and hormonal pathways [53]. However, findings remain inconsistent across cohorts, and some analyses report minimal or no subtype‐specific variation [31].

Similarly, differences between premenopausal and postmenopausal women remain unresolved. Postmenopausal women with diabetes frequently exhibit greater adiposity and elevated oestrogen exposure, which may explain stronger risk associations in this group [12]. Yet other studies have not replicated this pattern, raising questions about population diversity, study power and analytical approaches.

### 6.5 Medication‐Related Controversies: Metformin, Insulin and Cancer Risk

The potential influence of antidiabetic medications on breast‐cancer risk and prognosis represents one of the most contested topics in this field. Observational research has reported that metformin may confer protective or survival benefits, whereas insulin therapy has occasionally been associated with higher cancer risk [38, 39]. Published literature discusses these findings while stressing that many such studies are limited by confounding by indication; patients receiving insulin often have longer‐standing or more severe diabetes, which itself may be linked to poorer outcomes [9].

Randomized clinical‐trial evidence remains scarce and inconclusive. Some trials evaluating metformin as an adjunct to breast‐cancer treatment have produced neutral or modest effects on tumour outcomes, challenging earlier enthusiasm derived from observational signals [54]. Meta‐analyses of randomized clinical trials have similarly reported no clear survival benefit associated with metformin use in breast cancer patients [55]. These discrepancies fuel ongoing debate about whether medication effects are biological or whether they simply reflect underlying metabolic health and treatment patterns.

### 6.6 Generalizability Across Populations and Settings

A further point of controversy concerns the generalizability of existing evidence across different populations, ethnic groups and healthcare systems. Much of the research synthesized in Xiong et al. [13] and related reviews originates from North American and European cohorts. Differences in genetic background, environmental exposures, diet and healthcare access may influence the diabetes‐breast‐cancer relationship in other regions, including Asia, Africa and South Asia.

Limited data from low‐ and middle‐income countries constrain the ability to develop globally applicable risk‐prediction models or screening recommendations. This gap contributes to uncertainty about whether diabetes should be considered a universal risk modifier or a context‐dependent factor shaped by social and environmental determinants.

### 6.7 Interpretation of Prognostic Effects: Biology vs. Care Inequities

While many studies demonstrate worse survival among breast‐cancer patients with diabetes, debate persists regarding whether these outcomes reflect biological aggressiveness or inequities in care delivery. Reduced likelihood of receiving guideline‐concordant therapy, lower chemotherapy tolerance and delays in treatment initiation have been documented in some diabetic populations [19]. These system‐level factors may partially account for excess mortality.

### 6.8 Synthesis of Debates

The controversies surrounding diabetes and breast cancer illustrate that the association is multifaceted, heterogeneous and context‐dependent. Disagreement does not undermine the evidence base; rather, it highlights the complexity of metabolic‐oncologic interactions and underscores the importance of methodologically robust, multidisciplinary research. Future investigations must incorporate:

- Precise metabolic phenotyping,
- Longitudinal biomarkers of insulin and inflammation,
- Stratification by tumour subtype and menopausal status, and
- Evaluation of healthcare‐system determinants of outcome.

By addressing these uncertainties, future research can clarify causal pathways, refine risk stratification and inform more targeted prevention and management strategies.

## 7 Future Directions

### 7.1 Overview of Outstanding Knowledge Gaps

Despite growing evidence linking diabetes with breast‐cancer risk and outcomes, significant uncertainties remain regarding causality, biological mechanisms, risk stratification and optimal clinical strategies. Most available research is observational, relies on heterogeneous definitions of diabetes and metabolic status, and often lacks detailed biological or longitudinal data [8, 13]. Addressing these limitations will be essential for improving risk stratification, prevention strategies and therapeutic decision‐making in women affected by both conditions. Major research gaps and priority areas are highlighted in Table 2. The strategies outlined in this section—including biomarker development, metabolic phenotyping and intervention trials of antidiabetic agents—should therefore be regarded as research priorities and hypotheses to be tested, rather than established standards of care.

**TABLE 2:** Major research gaps and priority areas in studies examining diabetes and breast cancer.

Research gap	Current limitation	Priority area for future research
Establishing causality	Predominance of observational studies limits causal inference	Longitudinal cohorts integrating metabolic biomarkers and genetic susceptibility
Glycemic control and disease duration	Inadequate stratification by HbA1c levels and duration of diabetes	Prospective studies assessing dose–response relationships
Tumour molecular heterogeneity	Limited subtype‐specific analyses	Studies stratified by ER/PR/HER2 status and molecular signatures
Effects of antidiabetic therapies	Confounding by indication in observational analyses	Randomized trials evaluating metformin and newer antidiabetic agents
Global evidence gaps	Underrepresentation of low‐ and middle‐income populations	Population‐based studies in diverse geographic and socioeconomic settings

### 7.2 Need for Longitudinal and Mechanistically Informed Cohort Studies

Future research should prioritize large, prospective, multi‐ethnic cohort studies incorporating serial measurement of metabolic, hormonal and inflammatory biomarkers. Existing studies rarely capture trajectories of insulin resistance, glycemic control, adipokines, or IGF‐axis activity across time [19]. Without these data, it remains difficult to determine whether diabetes‐related metabolic alterations precede tumour development or simply coexist at diagnosis.

Integration of biospecimen repositories, metabolomics and transcriptomic signatures could clarify how specific metabolic phenotypes, such as hyperinsulinemia, chronic inflammation, or visceral adiposity, modify tumour initiation and progression [56]. Stratified analyses by tumour subtype, menopausal status and body‐composition profile are also essential to understand heterogeneity in risk and prognosis.

### 7.3 Inflammatory‐Oxidative‐Angiogenic Axes Beyond Diabetes

Chronic low‐grade inflammation, oxidative stress and pro‐angiogenic signalling represent convergent biological axes that are not unique to diabetes but also characterize other chronic conditions such as metabolic syndrome, non‐alcoholic fatty liver disease and cardiovascular disease [12, 13]. Future studies should therefore examine whether similar cytokine profiles (e.g., IL‐6, TNF‐α), NF‐κB–dependent transcriptional programmes, and oxidative stress signatures are associated with breast‐cancer risk and progression across these comorbidities, and whether they interact with, or act independently of, hyperglycemia and hyperinsulinemia [12, 36]. Multicenter cohorts incorporating detailed phenotyping of inflammatory and oxidative biomarkers could clarify whether a shared ‘inflammometabolic’ endotype underlies the clustering of diabetes, other chronic diseases, and more aggressive breast‐cancer behaviour [35, 56]. Such work would also help determine whether therapeutic strategies targeting inflammation or angiogenesis in diabetes are generalizable to broader high‐risk populations.

### 7.4 Development of Biomarkers and Risk‐Prediction Models

There is growing interest in developing precision‐risk tools that incorporate diabetes‐related markers into breast cancer prediction models. Current risk algorithms rely heavily on reproductive and genetic factors and rarely account for metabolic parameters such as fasting insulin, HbA1c, lipid patterns, or inflammatory markers.

From a translational perspective, there is a need to develop and validate biomarker panels that capture chronic inflammation and oxidative stress in women with diabetes who are at risk for breast cancer. Candidate markers include systemic inflammatory mediators such as high‐sensitivity C‐reactive protein (hs‐CRP), IL‐6, TNF‐α, and composite inflammatory indices, as well as oxidative‐stress indicators such as malondialdehyde, F2‐isoprostanes and measures of total antioxidant capacity [45, 46]. Integrating these measures with established metabolic indicators (e.g., HbA1c, waist circumference, lipid profiles) into risk‐prediction models may enable more precise identification of high‐risk subgroups and guide the intensity of breast‐cancer surveillance or preventive interventions among women with diabetes [13]. Prospective studies that repeatedly measure inflammatory and oxidative biomarkers before and after breast‐cancer diagnosis, and during survivorship, could clarify how changes in inflammatory and oxidative burden relate to treatment response, toxicity and long‐term outcomes in this population [45].

Future work should test whether integrating such biomarkers improves risk discrimination and clinical decision‐making, particularly for postmenopausal and obese women. Identification of high‐risk metabolic phenotypes may enable tailored prevention strategies, risk‐adapted surveillance strategies, or targeted lifestyle interventions.

Furthermore, tumour‐tissue studies examining IGF‐receptor expression, insulin‐receptor subtypes, and metabolic gene signatures may help define biologically distinct tumour subsets that arise in diabetic environments.

### 7.5 Intervention Trials on Glycemic Control and Metabolic Modification

Although observational studies suggest poorer outcomes among women with uncontrolled diabetes, causal evidence remains limited. Future randomized or pragmatic trials should evaluate whether improving glycemic control, weight status, diet quality and physical activity leads to measurable reductions in cancer recurrence or mortality.

In addition, trials examining metformin, GLP‐1 receptor agonists, SGLT2 inhibitors, and lifestyle interventions in breast‐cancer populations could help determine whether metabolic improvement itself provides oncologic benefit, independent of traditional cancer therapies [57].

### 7.6 Exploring Therapeutic Targeting of Metabolic‐Oncogenic Pathways

Mechanistic research suggests multiple opportunities for therapeutic targeting of pathways shared between diabetes and breast cancer, including PI3K/Akt/mTOR, AMPK activation, inflammatory signalling and adipokine pathways. Future translational research should examine how modulation of these pathways influences tumour biology, treatment response and resistance mechanisms.

Preclinical and early‐phase clinical studies may help identify combination strategies that pair endocrine or targeted cancer therapies with metabolic agents such as metformin or AMPK activators. Understanding which patient subgroups derive the greatest benefit will be essential to advancing precision‐medicine approaches.

### 7.7 Health‐System and Implementation Research

Another important direction involves studying how health‐system factors shape outcomes among women with diabetes and breast cancer. Delayed diagnosis, undertreatment and fragmented care may worsen prognosis in diabetic populations [22]. Future research should evaluate:

- Models of integrated oncology‐diabetes care,
- Interventions to improve screening participation,
- Culturally tailored patient‐navigation and education programmes, and
- Strategies to reduce treatment disparities in resource‐limited settings.

Implementation‐science frameworks will be particularly valuable for assessing feasibility, scalability and sustainability of such interventions across diverse health‐care environments.

### 7.8 Expanding Research in Low‐ and Middle‐Income Countries

Global evidence remains disproportionately concentrated in high‐income countries. Rising prevalence of both diabetes and breast cancer in Asia, Africa and South Asia underscores the need for context‐specific research that reflects local metabolic patterns, environmental exposures and health‐system realities [2].

Establishing regional cancer registries, metabolic‐oncology cohorts, and collaborative research networks will be crucial to generating equitable, globally relevant knowledge and guiding policy in low‐resource settings.

### 7.9 Synthesis and Forward‐Looking Perspective

Collectively, future directions emphasize the need for integrated, multidisciplinary research that links metabolic science with cancer biology and real‐world clinical practice. Advancing this field will require:

- Mechanistically informed longitudinal studies,
- Development of metabolic biomarkers and precision‐risk tools,
- Rigorous intervention and medication‐effect trials, and
- System‐level strategies to improve screening, treatment equity and survivorship care.

Such efforts may ultimately enable earlier detection, personalized prevention, and optimized outcomes for women living at the intersection of diabetes and breast cancer.

### 7.10 Chronic Infection, Idiopathic Granulomatous Mastitis and Diagnostic Complexity

Chronic infectious and immune‐mediated breast conditions warrant consideration in the diabetes‐breast‐cancer context. Idiopathic granulomatous mastitis (IGM) is a rare, benign chronic inflammatory disease of the breast that often presents with palpable masses, erythema, and radiologic features mimicking breast cancer, requiring histopathologic evaluation to exclude malignancy [58]. Some observational series and case reports suggest that IGM may be more frequent among women with diabetes or autoimmune disease, and concurrent IGM and breast cancer have been described in diabetic patients, highlighting potential intersections between metabolic dysregulation, chronic inflammation and diagnostic delay [59, 60]. Although IGM itself is not considered premalignant and does not appear to intrinsically increase breast‐cancer risk, future research should clarify whether chronic granulomatous mastitis or other persistent breast infections influence stage at diagnosis, imaging interpretation, treatment decision‐making, or survivorship outcomes in women with diabetes [58]. Multidisciplinary studies integrating radiology, histopathology, microbiology and immune‐metabolic profiling in such patients could illuminate how chronic infection‐associated inflammation fits within a broader translational framework linking metabolic disease, benign breast pathology and cancer care [58, 59].

## 8 Conclusion

There is a consistent relationship between diabetes mellitus, especially type 2 diabetes, and an increased risk of breast cancer and more clearly adverse outcomes following breast cancer diagnosis. Although the correlation seems to be at least in part due to common determinants including obesity and lifestyle factors, biologically plausible pathways suggest an independent contribution of metabolic disturbances related to diabetes. Hyperinsulinemia and IGF‐axis activation, chronic inflammation, oxidative stress, and altered sex‐hormone signalling provide pathways that may independently affect tumour initiation and progression as well as treatment tolerance. However, because most available data are observational and subject to residual confounding and reverse causation, diabetes should be viewed at present as a metabolic risk and prognostic modifier rather than a definitively established causal factor in breast carcinogenesis. Clinical issues, such as comorbidity burden, and possible screening differences and therapy‐related metabolic problems support the necessity to provide oncology and diabetes care integrally, including active screening compliance, active glycemic control in the context of treatment, and survivorship plans to consider cardiometabolic risks. The future research must focus on longitudinal cohort studies with repeated metabolic biomarkers, subtype analyses and pragmatic trials to assess the possibility of metabolic optimization and modern antidiabetic drugs to enhance cancer outcomes, especially in low‐ and middle‐income countries where the dual burden of diabetes is growing very fast. Finally, the inflammatory, oxidative, and angiogenic pathways highlighted in this review may represent a shared biological substrate linking not only diabetes but also other chronic diseases to breast‐cancer risk and prognosis, underscoring the need for cross‐disease translational research agendas that bridge metabolic medicine and oncology.

## Author Contributions

**Imran Hossain:** conceptualization, writing – review and editing. **Fatema‐Tuz‐Zohora:** conceptualization, writing – original draft, writing – review and editing. **Israt Jahan:** writing – review and editing, conceptualization. **Mohammad Harun‐Ur‐Rashid:** conceptualization, writing – review and editing.

## Funding

The authors have nothing to report.

## Conflicts of Interest

The authors declare no conflicts of interest.

## Data Availability

Data sharing not applicable to this article as no datasets were generated or analysed during the current study.
